# Overlapping Phenotypes of Compulsive Buying-Shopping Disorder and Borderline Personality Disorder: An Evidence-Based Model

**DOI:** 10.1016/j.abrep.2026.100669

**Published:** 2026-01-23

**Authors:** Nora M. Laskowski, Luisa Sabel, Gerrit Brandt, Georgios Paslakis

**Affiliations:** University Clinic for Psychosomatic Medicine and Psychotherapy, Medical Faculty, Campus East-Westphalia, Ruhr-University Bochum, Virchowstr. 65, 32312 Luebbecke, Germany

**Keywords:** Compulsive Buying-Shopping Disorder, Borderline Personality Disorder, Transdiagnostic, Psychological Mechanisms, Etiological Overlap, Model, Differential Diagnostic

## Abstract

**Background:**

Compulsive Buying-Shopping Disorder (CBSD) and Borderline Personality Disorder (BPD) are two clinically relevant conditions that frequently co-occur. Previous studies suggest overlapping etiological pathways, yet a structured comparison of the two disorders is missing. This paper aimed to map and contrast the key psychological constructs associated with CBSD and BPD based on established diagnostic criteria and the existing literature.

**Methods:**

A comprehensive, iterative literature search was conducted in PubMed and Google Scholar without publication date restrictions, including qualitative, quantitative, and review studies in English and German. Constructs were categorized based on the strength and consistency of the evidence, and findings were synthesized narratively and illustrated visually.

**Results:**

Shared constructs include impulsivity, affective/emotional stability and regulation, self-regulation/control, self-concept/identity, attachment style/emotional dependence, decision-making, perfectionism, and novelty seeking. For CBSD, constructs such as cue-reactivity/craving, compulsivity, behavioral inhibition/activation system, materialism, attachment to possessions, and approach-oriented personality traits were more prominent. In contrast, BPD-specific constructs include internal representations of self and others/object relations, mentalization, chronic emptiness, perceptual/interpretation bias, non-suicidal self-injurious behavior, social relationships, and dissociative symptoms.

**Conclusion:**

The resulting model offers a preliminary framework to distinguish shared and unique features of CBSD and BPD, supporting transdiagnostic understanding and contributing to improved diagnostic precision and intervention strategies.

## Highlights

• Narrative overview of overlapping and differential features of CBSD and BPD.
• Findings highlight key transdiagnostic mechanisms.
• Framework provides structure to differentiate shared/disorder-dominant constructs.

## 1 Background

Compulsive Buying-Shopping Disorder (CBSD) is characterized by urges and/or impulses to make purchases that are perceived as irresistible. When acted upon, these urges are associated with a loss of control over buying-shopping behavior, resulting in frequent episodes and excessive purchases of goods that are unnecessary in quantity and/or type and are often barely used or not used at all (Laskowski et al., 2021, McElroy et al., 1994; Müller, Laskowski, et al., 2021). CBSD has been described for more than 100 years (Demling and Müller, 2023, Magnan, 1892), but is still not listed as a distinct mental disorder in either the 11^th^ revision of the International Classification of Diseases (ICD-11; World Health Organization, 2022) or the 5^th^ edition of the Diagnostic and Statistical Manual of Mental Disorders (DSM-5; American Psychiatric Association, 2013). Nevertheless, research interest has increased substantially, and accumulating evidence supports its clinical relevance (e.g., Etxaburu et al., 2024, Laskowski et al., 2024, Marris, 2025, Müller et al., 2023, Müller et al., 2025, Redine et al., 2023, Thomas et al., 2024).

In contrast to CBSD, Borderline Personality Disorder (BPD) is an officially recognized mental disorder (American Psychiatric Association, 2013, World Health Organization, 2022) with an estimated point prevalence of 0.7% to 2.7% in adult community populations (Eaton and Greene, 2018, Ellison et al., 2018). BPD is characterized by emotional dysregulation, high rates of self-harm, affective instability, disturbed interpersonal relationships, and substantial psychiatric comorbidity (Lieb et al., 2004, Winsper et al., 2020).

Evidence on the co-occurrence of CBSD and BPD is mixed and appears to depend on the assessment methods and examined cohorts. Studies using screening instruments reported comparatively high rates of BPD, such as 37% among treatment-seeking individuals with compulsive buying^1^ (Schlosser et al., 1994), as assessed using the Personality Disorder Questionnaire–Revised (Hyler et al., 1987), and 26% in individuals with compulsive buying (Maraz et al., 2016), as assessed using the McLean Screening Instrument for Borderline Personality Disorder (Zanarini et al., 2003). An exception to these rather high prevalence rates is the study by Zander et al. (2016), which reported a markedly lower estimated BPD prevalence of 3.2% using the Borderline Symptom List–23 (BSL-23; Bohus et al., 2009) in a clinical outpatient sample with pathological buying. In contrast, studies employing structured clinical interviews rather than mere screening instruments yielded significant prevalence estimates, reporting BPD diagnoses in approximately 15% of treatment-seeking individuals with compulsive buying (Schlosser et al., 1994) and 20% in patients with pathological buying (Müller, Mühlhans, et al., 2009).

Beyond categorical diagnoses, dimensional symptom assessments provide more consistent support for an association between BPD and CBSD-related symptomatology. Sansone, Chang, Jewell, Sellbom et al. (2013) found moderate to strong correlations between BPD features –assessed with the Personality Diagnostic Questionnaire–4 (Hyler, 1994) and the Self-Harm Inventory (Sansone et al., 1998)- and compulsive buying severity. Similarly, Nicolai and Moshagen (2018) reported significant positive associations between pathological buying and BPD symptom severity assessed with the BSL–23 (Bohus et al., 2009).

Converging evidence at the behavioral level further supports an association between BPD and impulsive spending behaviors. Specifically, Selby et al. (2010) found that BPD pathology predicted impulsive spending as a form of behavioral dysregulation in women with anorexia nervosa, while Selby and Joiner (2013) identified impulsive shopping as one of several emotion-driven dysregulated behaviors associated with elevated BPD symptoms in student and community samples. Importantly, these findings refer to impulsive, situational buying-shopping behaviors.

Overall, available evidence suggests that overlap between CBSD and BPD may partly reflect shared etiological factors (Black, 2022, Claes and Müller, 2017, Laskowski and Müller, 2021, Laskowski and Müller, 2023 Müller, Mitchell, et al., 2009; Schlosser et al., 1994). At the same time, only a limited number of studies have explicitly examined CBSD-BPD comorbidity and related mechanisms, leaving both similarities and, importantly, distinctions insufficiently clarified. For instance, Maraz et al. (2016) modeled compulsive buying in individuals with BPD and identified impulsivity and contingent self-esteem as key predictors; however, the study did not systematically address conceptual overlap and boundaries between the two conditions.

While similarities and distinctions between CBSD and other psychiatric conditions (e.g., obsessive–compulsive disorder [OCD]), have been examined in greater detail (e.g., Grant and Chamberlain, 2024, Kim et al., 2018, Lawrence et al., 2014, Nicolai and Moshagen, 2017, Weinstein et al., 2015), a comparable, theory-driven comparison of CBSD and BPD is still largely lacking, despite recurring clinical observations of symptom overlap and diagnostic ambiguity. This distinction is clinically relevant. In BPD, reductions in dysregulated behaviors (e.g., binge eating, substance misuse) are considered key indicators of treatment success. Conversely, clinical reports in CBSD indicate symptom substitution and shifts in comorbid symptom patterns following reductions in buying-shopping behavior (Laskowski & Müller, 2021; Müller, Laskowski, et al., 2021). More generally, comorbid psychopathology substantially influences symptom severity, illness trajectory, and treatment response in pathological buying, often complicating prognosis and therapeutic outcomes (Müller et al., 2008).

Importantly, although CBSD and BPD may share certain dysregulated behaviors, existing evidence suggests that these behaviors reflect qualitatively different mechanisms and levels of severity rather than comparable structural impairments, particularly regarding identity disturbance, inhibitory control, and emotion regulation. This underscores the need for a structured comparative examination of CBSD and BPD that integrates shared and distinct symptom patterns, underlying mechanisms, and clinical implications. To our knowledge, no previous study has systematically contrasted CBSD and BPD in a theory-driven manner based on (proposed) diagnostic criteria and the existing literature. Therefore, the present paper aims to elucidate both overlapping and differing features of CBSD and BPD, thereby contributing to improved diagnostic precision, reduced risk of misdiagnosis, and the development of more targeted, disorder-specific interventions from a transdiagnostic perspective.

## 2 Methods

To identify associated phenotypic constructs (i.e., observable symptom dimensions and behavioral manifestations that may extend beyond formal diagnostic criteria), the proposed diagnostic criteria for CBSD (Müller, Laskowski, et al., 2021) were used; for BPD, the ICD-11 and DSM-5 criteria were applied. A literature search was conducted by NML, LS, and GB in PubMed and Google Scholar between March and October 2025.

The search followed a flexible and exploratory strategy and was iteratively refined throughout the manuscript development as relevant concepts, symptom domains, and theoretical perspectives emerged. Specifically, combinations of keywords and MeSH terms related to CBSD and BPD (e.g., “compulsive buying” OR “pathological buying” OR “buying-shopping disorder” AND “borderline personality disorder”) were applied. Additional terms were incorporated using backward and forward citation tracking (e.g., “impulsivity”, “materialism”, “dissociation”, “emptiness”).

No restrictions were placed on study design; qualitative and quantitative studies, including (systematic) reviews and meta-analyses, were considered. The search was not limited by publication date but was restricted to studies published in German or English. As the aim of this review was to provide a theory-driven comparative synthesis rather than a comprehensive systematic review, no predefined search protocol or fixed search string was applied.

**Fig. 1:**
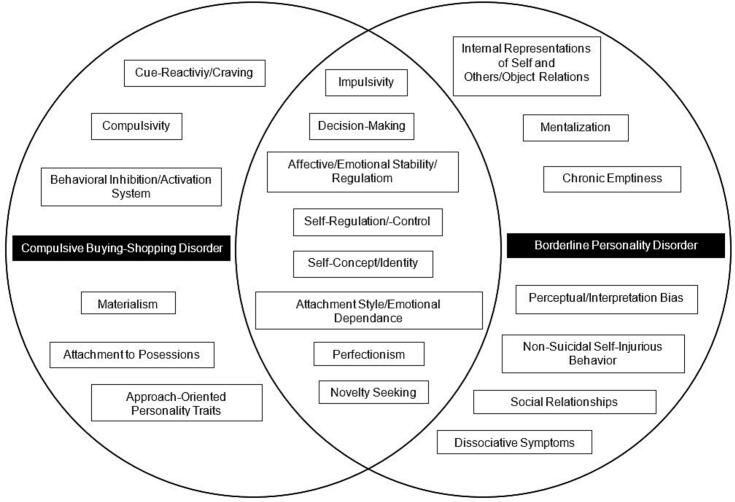
A Framework Representation of Compulsive Buying-Shopping Disorder and Borderline Personality Disorder. Note. The order of constructs is arbitrary and does not imply hierarchy or prioritization. Construct allocation is based on literature-derived evidence and does not indicate exclusivity to either condition. The framework is not intended to be exhaustive but should be understood as a dynamic, evolving model that may be refined as further empirical evidence emerges. All studies informing the Results section are listed in Supplementary Table 1.

Identified constructs for CBSD and BPD were synthesized narratively by NML, LS, and GB through comparative evaluation of their definition, conceptual relevance, and empirical support in the literature and were visually summarized (Fig. 1). References presented in the table serve an illustrative function within this integrative framework and do not represent an exhaustive or systematically selected set of eligible studies. To increase transparency regarding the empirical basis of the proposed framework, all publications that informed the identification and evaluation of constructs in the Results section are provided in Supplementary Table 1.

Construct allocation was based on converging patterns across the broader literature rather than on single studies alone. Consistent with a scoping-oriented approach, this synthesis aimed to map key concepts, characterize the breadth of available evidence, and examine how constructs are defined and operationalized in the literature.

In a final analytic step, all identified constructs were cross-checked against the literature on both CBSD and BPD to ensure comprehensive coverage and to verify their empirical support across disorders. This cross-checking procedure was intended to evaluate constructs beyond a single disorder-specific body of literature and to reduce the risk of selective citation or overrepresentation of disorder-focused perspectives, while acknowledging that relevant evidence may nevertheless have been missed.

Allocation of constructs to CBSD, BPD, or both was based on qualitative appraisal of the relative strength and volume of available evidence, including consistency of findings, methodological rigor, construct relevance, and replication across independent samples. This appraisal did not rely on a formal scoring system but was explicitly informed by the research team’s clinical expertise in CBSD and BPD and its extensive experience in conducting systematic and narrative reviews as well as domain-specific research. Team members with established research backgrounds in CBSD (here NML) and clinical and research expertise in BPD (here GP), including specialized inpatient care, contributed to the integrative evaluation and construct allocation.

The presentation of constructs followed a consistent comparative logic: overlapping constructs were examined with disorder-specific evidence for both CBSD and BPD within a joint framework to facilitate direct comparison, whereas constructs predominantly associated with one disorder were discussed separately. Where disorder-specific evidence was sparse or absent, this was explicitly stated and contextualized within the respective section to avoid overinterpretation.

The classification of constructs does not imply exclusivity; rather, several constructs may occur in the context of both conditions. Accordingly, the proposed framework should be understood as a dynamic and hypothesis-generating, open to refinement as further empirical evidence emerges.

## 3 A Framework Representation of Compulsive Buying-Shopping Disorder and Borderline Personality Disorder

### 3.1 Overlapping Constructs

In the following, overlapping phenotypic constructs of CBSD and BPD are examined within a joint comparative framework. Disorder-specific evidence for both conditions is presented to facilitate direct comparison, as summarized in Table 1 and visually represented in Fig. 1. The order of the constructs is arbitrary and does not imply any ranking or hierarchy.

**Table 1:** Overlapping features of Compulsive Buying-Shopping Disorder and Borderline Personality Disorder

**Definition**	**CBSD relevance**	**BPD relevance**
*Impulsivity*		
“Predisposition toward rapid, unplanned reactions to internal or external stimuli without regard to the negative consequences of these reactions” (Evenden, 1999)	• “Repetitive impulses to buy/shop” in the diagnostic criteria by Müller, Laskowski et al. (2021) • Included in the ICD-11 as an example of “other specified impulse control disorders” (World Health Organization, 2022) • Impulsivity is positively associated with compulsive buying severity (Davenport et al., 2012), with urgency and lack of premeditation being particularly relevant facets of impulsivity (Billieux et al., 2008) • Clinical and neuropsychological findings indicate elevated impulsivity-related traits, while global executive control and response inhibition deficits are inconsistent in CBSD (Black et al., 2012)	• Diagnostic criterion in DSM-5 (American Psychiatric Association, 2013) and ICD-11 (World Health Organization, 2022) as tendency to act rashly in states of high negative affect • “Cold” (emotionally neutral) impulse control is less impaired in BPD, while “hot” (emotionally charged) impulse control is more consistently affected (Sebastian et al., 2013) • Response inhibition shows minimal disturbance, cognitive and interference inhibition findings are mixed, but decisional and motivational impulse control are reliably impaired in BPD (Sebastian et al., 2013) • Individuals with BPD struggle with impulse control when experiencing strong emotions because of dysfunction in certain brain networks, particularly involving an overactive amygdala and its impact on prefrontal areas that help regulate behavior (Soloff et al., 2017)
*Decision-Making*		
Decision-making refers to the process of selecting an option from a range of possible alternatives, guided by the individual’s personal values, goals, and preferences (Harris, 1998). The concept of decision-making encompasses processes such as making choices under conditions of uncertainty or risk, delay discounting, information sampling, and reinforcement reversal learning (Jones et al., 2019).	• Experimental studies yield mixed findings regarding decision-making deficits in CBSD. Disadvantageous decision-making is primarily observed under ambiguity and in buying-shopping-related contexts, whereas performance under explicit risk conditions appears largely preserved (Derbyshire et al., 2014, Lo and Harvey, 2012, Nicolai and Moshagen, 2017, Trotzke et al., 2015) • Maladaptive attitudes toward decision-making, specifically fears about making decisions, were significantly associated with compulsive buying symptom severity (Kyrios et al., 2004) • More egoistic everyday moral decision-making styles moderated the relationship between CBSD symptoms and ego-oriented, buying/shopping-related decisions (Müller et al., 2022)	• Individuals with BPD display a preference for immediate over delayed rewards (Paret et al., 2017) • Individuals with BPD exhibit impaired performance on the IGT compared to controls as they tend to select more disadvantageous options (Bechara et al., 1994) • BPD patients were more likely to take risks when the probability of winning was low, and were more risk-averse when the probability of winning was high compared to controls (Bajzát et al., 2023)
*Affective Regulation and Emotional Stability*		
• Affective regulation: processes through which individuals attempt to influence the emotions they experience, their timing, and how they are felt or expressed (Gross, 1998, Gross, 2015) • Emotional Stability: Inability to manage or respond to emotional experiences in a healthy way (Gratz & Roemer, 2004)	• It can be a strategy to “relieve negative mood (e.g., nervousness, tension, negative feelings and thoughts, discomfort, boredom)” (Müller, Laskowski, et al., 2021), reflecting cue mediated mood regulation rather than persistent affective instability or global emotion regulation deficits • CBSD can also be driven by the pursuit of generating or chasing “positive mood (e.g., pleasure, excitement)” (Müller, Laskowski, et al., 2021) • Individuals with compulsive buying seem to use their buying-shopping behavior as a way to cope with both negative and positive emotional states (Claes and Müller, 2017, David et al., 2024, Kyrios et al., 2004) • Compulsive buying/shopping is described to provide only short-term emotional relief, leading to long-term negative consequences (David et al., 2024)	• Diagnostic criteria in ICD-11 (World Health Organization, 2022) and DSM-5 (American Psychiatric Association, 2013); described as a result of marked mood reactivity (e.g., severe episodic dysphoria, irritability or anxiety) • Affective instability in individuals with BPD is marked by more intense negative emotions (Selby et al., 2009), wider range of negative feelings, frequent and sudden mood swings linked to specific emotional states, emotional responses partly triggered by external events, severity of negative mood as a potential risk factor for suicide (Nica & Links, 2009) • Emotional dysregulation in BPD is closely linked to emotion-dependent failures of behavioral control (Soloff et al., 2017)
*Self-Concept and Identity*		
The structured combination of an individual’s traits, attitudes, self-knowledge, cognitive patterns, self-representations, social roles, and relationships (Guenther et al., 2016)	• Perceived identity “gains” were directly related to compulsive buying, reflecting compensatory self-enhancement through consumption rather than structural identity disturbance (Dittmar, 2005a) • Identity confusion was associated with buying-shopping disorder symptoms, primarily at the level of reduced self-concept clarity and self-definition, rather than pervasive identity instability (Claes et al., 2016, Müller et al., 2020) • Compulsive buying is linked to materialistic value endorsement and self-discrepancies (i.e., gaps between the actual and ideal self), indicating compensatory regulation of self-esteem and self-concept uncertainty (Dittmar, 2005a, Noguti and Bokeyar, 2014, Sharif and Khanekharab, 2017)	• Core symptom as “identity disturbance: markedly and persistently unstable self-image or sense of self” (American Psychiatric Association, 2013) and “disturbances in self-image, aims, and internal preferences” (World Health Organization, 2022) • Faggioli et al. (2024) demonstrated a link between a fragmented self-image and impaired autobiographical coherence, and a disrupted experience of time in BPD • Although some aspects of identity disturbance are linked to a history of sexual abuse, it remains a distinguishing feature of BPD regardless of such history (Wilkinson-Ryan & Westen, 2000)
*Self-Regulation and -Control*		
Self-regulation can be defined as the process by which individuals attempt to modify their internal states or responses, including the regulation of thoughts, emotions, impulses, appetites, task performance and attentional processes (Baumeister & Vohs, 2004)	• In the proposed diagnostic criteria, the persistent and recurrent experience of diminished control over buying-shopping is defined as a core feature of CBSD (Müller, Laskowski, et al., 2021). This subjective loss of control has been linked to elevated impulsivity and emotion-driven responding (Billieux et al., 2008) • Evidence regarding generalized self-regulatory and inhibitory control deficits in CBSD is mixed and does not allow for unequivocal conclusions. Experimental studies indicate preserved or context-dependent self-regulation and inhibitory control, suggesting facet- and situation-specific impairments rather than global deficits (Billieux et al., 2010; Vogt et al., 2015). In contrast to BPD, these findings do not support pervasive or trait-like self-regulatory deficits comparable to those observed in BPD • Dual-pathway models propose that self-regulatory difficulties in addictive behaviors primarily emerge under conditions of heightened emotional or reward-driven activation, rather than reflecting stable control deficits (Van Malderen et al., 2024) • Positive associations have been reported between CBSD symptoms and deficits in stimulus interference control, as measured by a Stroop Matching Task, indicating selective impairments in suppressing task-irrelevant stimuli (Lindheimer et al., 2020)	• A core feature of BPD is the lack of effective self-regulatory mechanisms, such as pronounced emotional dysregulation, which often manifests in everyday social contexts (King-Casas et al., 2008) • Low self-control in individuals with BPD contributes to significant interpersonal (Euler et al., 2021) and behavioral difficulties, including engagement in risk behaviors (Johnson et al., 2017) • The enhancement of regulatory capacities is recommended as a key focus in therapeutic interventions for BPD (Bud et al., 2023) • In individuals with BPD, self-report data indicate poor self-regulation in daily-life activities, along with extreme scores on personality traits compared to healthy controls (Lozano et al., 2016) • BPD is associated with impairments in interference control, particularly in managing interference arising from memory (Lozano et al., 2016)
*Attachment Style and Emotional Dependence*		
In both disorders, insecure attachment styles are implicated, albeit with different functional roles and clinical significance. Anxious-preoccupied attachment is described as an insecure attachment style defined by a strong desire for intimacy and a persistent fear of rejection or abandonment (Bartholomew & Horowitz, 1991)	• Emotional dependence is a significant positive predictor of buying-shopping disorder and a preoccupied attachment style indirectly increases buying-shopping disorder risk through emotional dependence (Etxaburu et al., 2024) • Anxious attachment is linked to excessive acquiring behavior like excessive buying, and this relationship is influenced by distress intolerance and the tendency to humanize objects for emotional comfort (Norberg et al., 2018) • Among the insecure attachment patterns, especially the fearful attachment style showed a significant and positive association with compulsive online shopping behavior (Topino et al., 2022)	• Both attachment anxiety and attachment avoidance were correlated with BPD, with a stronger correlation between BPD traits and attachment anxiety than with attachment avoidance (Smith & South, 2020) • Individuals with low personal agency (extent to which an individual feels they can influence or direct their own life) were more likely to have higher BPD symptoms and insecure attachment styles, especially fearful and preoccupied (Hashworth et al., 2021) • Individuals with BPD were significantly more to report low personal agency and insecure attachment compared to those without (Hashworth et al., 2021) • Elevated levels of rejection sensitivity were moderately correlated with increased BPD symptomatology (Gao et al., 2017)
*Perfectionism*		
Perfectionism includes self-oriented (striving for personal perfection), other-oriented (expecting perfection from others), and socially prescribed forms (feeling pressured by others' expectations and fear of criticism) (Hewitt & Flett, 1991)	• In CBSD, perfectionism appears primarily as a self-regulatory and self-evaluative vulnerability rather than a core personality feature • Internalized perfectionist standards are associated with compulsive and impulsive buying (Martinelli et al., 2014) • Individuals with high perfectionistic expectations may attempt to achieve unrealistic levels of social acceptance or exert control over their impulses or possessions (Kyrios et al., 2004)	• Perfectionism is associated with the severity of BPD (Dimaggio et al., 2018) • Socially prescribed perfectionism has been identified as particularly relevant to BPD (Stoeber, 2014) as it predicted an increase in manifestations of BPD over a three-month period (Chen et al., 2019)
*Novelty Seeking*		
Novelty seeking is a personality trait marked by a tendency toward excitement in response to new experiences, impulsivity, and a preference for variety and change (Cloninger et al., 1994).	• Research reported positive correlations between novelty seeking and pathological/compulsive buying symptoms (Claes and Müller, 2017, Di Nicola et al., 2010, Fernández-Aranda et al., 2019, Jiménez-Murcia et al., 2015). • Individuals with CBSD exhibited a stronger tendency to seek novel and stimulating experiences compared to controls (Black et al., 2012). • Gender differences in novelty seeking have been observed, with women scoring higher than men (Machado et al., 2022).	• BPD patients differ significantly from control groups in levels of novelty seeking (Fossati et al., 2001), with men generally showing higher levels than women (Barnow et al., 2007, Bozzatello et al., 2024). • Elevated novelty seeking, particularly in the context of childhood trauma and adolescent psychopathology, may play a critical role in the early development of BPD (Joyce et al., 2003). • High harm avoidance and novelty seeking, along with low reward dependence, emerged as key vulnerability factors in adolescents with BPD (Kaess et al., 2013).

Note. The order of constructs is random. For consistency with the ICD-11 framework, we use the term Compulsive Buying-Shopping Disorder (CBSD), while retaining the original terminology used in individual studies when referring to specific findings. BPD = Borderline Personality Disorder, WHO = World Health Organization, APA = American Psychiatric Association, IGT = Iowa-Gambling Task. References listed illustrate representative empirical patterns and do not constitute an exhaustive or systematically selected evidence base

Across the reviewed literature, overlapping constructs between CBSD and BPD primarily cluster around impulse-driven behavior, affective modulation, and self-referential regulatory processes. Importantly, these overlaps are predominantly functional rather than structural in nature and should not be interpreted as reflecting comparable levels of personality pathology or regulatory impairment across disorders.

### 3.2 Constructs Predominantly Associated With Compulsive Buying-Shopping Disorder

The constructs with greater empirical support in the context of CBSD described here are shown on the left-hand side of Fig. 1. This does not imply that these constructs are entirely absent in BPD; rather, available empirical evidence is currently more consistent and specific for CBSD.

#### 3.2.1 Craving and Cue-Reactivity

Craving–the irresistible desire to engage in a behavior, often triggered by cue-reactivity (Tiffany & Wray, 2012)–is a core feature of addictive disorders in DSM-5 (American Psychiatric Association, 2013) and ICD-11 (World Health Organization, 2022) and is reflected in the proposed CBSD criteria as “craving for the high or for relief while buying/shopping” (Müller, Laskowski, et al., 2021). In CBSD, craving is implicated in cue-dependent decision-making: individuals with stronger craving reactions or higher symptom severity show more disadvantageous decisions when exposed to online buying-shopping cues (Trotzke et al., 2019), and cue-induced craving differentiates CBSD from controls (Trotzke et al., 2014). Neuroimaging findings further indicate stronger neural responses to buying-shopping cues with increasing CBSD severity (Trotzke et al., 2021). In BPD, craving-like experiences have been discussed mainly in relation to specific urges (e.g., self-harm) and appear behavior-specific and context-dependent rather than a transdiagnostic hallmark (Elango et al., 2025, Victor et al., 2012).

#### 3.2.2 Compulsivity

Compulsivity is characterized by repetitive, habitual behaviors, either visible or mental, that lack adaptive purpose and are carried out rigidly or to prevent perceived negative outcomes, often leading to functional impairment (Fineberg et al., 2014).

Empirical findings in CBSD point to compulsivity-like features such as repetitive buying urges, diminished control, and perseverative decision-making. However, evidence for core compulsivity mechanisms —particularly habit formation or a shift from goal-directed to habitual responding— remains limited and inconsistent (Theisejans et al., 2025). Accordingly, compulsivity-related phenomena in CBSD are currently better conceptualized as component processes embedded within broader impulsive–addictive dynamics rather than as a fully developed compulsive profile.

Historically, CBSD was conceptualized along an OCD-spectrum, with both impulsivity and compulsivity considered relevant (Christenson et al., 1994, Kellett and Bolton, 2009). This is reflected in current classifications, where CBSD is referenced in relation to excessive acquisition within hoarding-related disorders in both ICD-11 and DSM-5 (American Psychiatric Association, 2013, World Health Organization, 2022).

Elevated rates of CBSD have been reported in OCD samples, and substantial overlap with hoarding behavior has been documented (Frost et al., 2002, Lejoyeux et al., 2005, Müller et al., 2007), although hoarding severity does not appear to systematically index CBSD severity (Varvaras et al., 2025).

Within contemporary addiction frameworks such as the Interaction of Person-Affect-Cognition-Execution (I-PACE) model, compulsive elements are conceptualized as part of a broader interaction between reinforcement learning, impulsivity, and executive control deficits (Brand et al., 2019, Brand et al., 2025). In CBSD, compulsive features (e.g., repetitive intrusive buying-related thoughts) are therefore embedded within a complex constellation of interacting mechanisms rather than constituting an isolated compulsivity construct (Müller, Laskowski, et al., 2021).

In BPD, compulsivity-like phenomena have mainly been discussed in relation to repetitive non-suicidal self-injury or other maladaptive behaviors. However, available evidence suggests that these behaviors reflect context- and frequency-dependent regulatory strategies rather than structural compulsivity as observed in obsessive–compulsive spectrum disorders (Lutz et al., 2022). Consequently, direct evidence for a coherent compulsivity construct comparable to CBSD or OCD is currently lacking in BPD.

#### 3.2.3 Behavioral Inhibition and Behavioral Activation System

Gray’s Reinforcement Sensitivity Theory (Gray, 1970, Gray, 1982) describes two core motivational systems: the Behavioral Inhibition System (BIS), which responds to cues of punishment, non-reward, extreme novelty, and innate fear, and the Behavioral Activation System (BAS), which regulates approach behavior in response to appetitive stimuli and is sensitive to signals of reward and non-punishment.

In CBSD, converging evidence points to heightened BAS sensitivity, particularly within the impulsivity-related BAS components. Elevated BAS activity has been reported especially in women with CBSD (Claes et al., 2011, Claes and Müller, 2017, Voth et al., 2014) and is consistent with reward-driven approach tendencies that may increase vulnerability to strong urges to buy/shop. This is reflected in the proposed diagnostic criteria for CBSD, which include a “strong desire or irresistible urge to engage in buying/shopping activities” (Müller, Laskowski, et al., 2021).

In addition, heightened BIS sensitivity —reflecting increased punishment sensitivity and avoidance tendencies— has been discussed as a vulnerability factor in CBSD, particularly among men, by amplifying anxiety and stress and thereby reinforcing buying-shopping as a maladaptive coping strategy (Claes and Müller, 2017, Verplanken and Sato, 2011).

BIS/BAS sensitivity has also been examined in BPD, with findings indicating heightened reward sensitivity, elevated BAS activity, and concurrent hyperreactivity of both BIS and BAS systems (Berenson et al., 2021, Bijttebier et al., 2009, Ross et al., 2013). These patterns have been interpreted as reflecting the characteristic co-occurrence of impulsivity and anxiety in BPD, and BIS/BAS sensitivity has been shown to mediate associations between BPD features and broader psychopathological symptoms (Bilge & Emiral, 2022).

Importantly, the available literature does not allow for direct quantitative or mechanistic comparisons of BIS/BAS sensitivity between CBSD and BPD. Existing findings indicate disorder-specific patterns of motivational dysregulation that differ in functional role, contextual embedding, and clinical significance. To date, no studies have systematically examined BIS/BAS sensitivity in BPD in a manner that allows direct comparison with CBSD-specific findings. Accordingly, BIS/BAS alterations should be understood as functionally relevant within each disorder, rather than as evidence of comparable underlying motivational pathology.

#### 3.2.4 Materialism

Materialism encompasses a set of beliefs reflecting how strongly individuals value, idealize, and attach meaning to material possessions (Kellett & Bolton, 2009).

A robust and consistent finding among individuals affected by CBSD is a pronounced material value orientation (Laskowski et al., 2021; Müller, Laskowski, et al., 2021). Materialistic value orientations, particularly when linked to ideal-self motives, have been shown to increase vulnerability to CBSD (Claes et al., 2016). This association persists when CBSD is compared with other behavioral addictions, such as social network use disorder; (Wegmann et al., 2023), and extends beyond clinical populations, with significant correlations also observed in general population studies (Müller et al., 2020, Villardefrancos and Otero-López, 2016). More recent findings further indicate that ego-oriented, self-focused buying-shopping decisions are associated with both higher endorsement of materialistic values and greater CBSD symptom severity (Müller et al., 2022). Importantly, materialism appears to function as a mediating mechanism linking identity-related vulnerabilities to CBSD (Claes et al., 2016).

In contrast, materialism has not been systematically examined as a core construct in BPD, and direct empirical evidence supporting its relevance for BPD is currently lacking.

#### 3.2.5 Attachment to Possessions

Ball and Tasaki (1992) define attachment to possessions as the degree to which an individual incorporates objects into their cognitive representation of the self, using them to develop and maintain their self-concept.

A key characteristic of CBSD is the “excessive purchasing of items without utilizing them for their intended purposes” (Müller, Laskowski, et al., 2021). From a cognitive-affective perspective, dysfunctional beliefs and emotional attachments to possessions play a crucial role in the development and maintenance of CBSD (Frost et al., 2007; Müller, Claes, et al., 2021). These include beliefs that buying-shopping alleviates negative emotional states, overvaluation of the uniqueness or irreplaceability of desired items, and fears of missing out on purchasing opportunities (Kyrios et al., 2004). Insecure, but not secure, object attachment has been specifically associated with symptoms of hoarding and compulsive buying (David & Norberg, 2022).

Dysfunctional possessions-related cognitions appear to function as a mechanism for regulating self-concept and emotional states, hereby reinforcing the compulsive acquisition cycle (Moulding et al., 2017). Consistent with this view, Frost et al. (2007) found that high levels of self-ambivalence and attachment insecurity were significantly associated with CBSD symptom severity in women. These findings align with broader evidence suggesting that compulsive shopping may serve as a maladaptive strategy to reduce discrepancies between actual and ideal self (Dittmar, 2005b). Early studies further indicate gender-specific patterns in emotional investment in consumer goods, with women more likely to purchase emotionally and symbolically meaningful items (e.g., clothing, shoes), whereas men more often acquire functional or recreational products (e.g., electronics, sports equipment) (Dittmar et al., 1995, Scherhorn et al., 1990).

In contrast, object-related attachment in BPD has primarily been discussed in relation to transitional objects that serve affect-regulatory and interpersonal functions, rather than attachment to possessions acquired through excessive buying or material accumulation (Cardasis et al., 1997). Although object attachment has been linked to hoarding-related phenomena, direct empirical evidence for possession-focused attachment as conceptualized in the CBSD literature is currently lacking in BPD (Timpano & Port, 2021).

#### 3.2.6 Approach-Oriented Personality Traits

Recognition, Attention, and/or Arousal Seeking were subsumed under the umbrella term Approach-Oriented Personality Traits, as they share a heightened tendency to approach rewarding or stimulating cues. Approach motivation refers to behavior guided by the anticipation of a positive or desirable outcomes (Elliot & Thrash, 2002). Attention seeking describes behaviors aimed at attracting others’ attention, often persistent and perceived as disruptive over time (Mellor, 2005). Arousal seeking reflects an individual’s tendency to pursue novel, complex, or stimulating situations to maintain or increase their internal arousal (Mehrabian & Russell, 1973).

In the context of CBSD, early conceptual work suggests that individuals may view the acquisition of financial and material resources as a means to address emotional and interpersonal needs, including the need for recognition (Hanley and Wilhelm, 1992, Kellett and Bolton, 2009). Empirically, patients with CBSD often report excessive buying-shopping of appearance-related and status-oriented products, based on the belief that such purchases help conceal perceived personal flaws and elicit admiration or affection from others (Müller, Claes, et al., 2021). This pattern appears more pronounced in women, consistent with earlier findings showing greater purchasing of self-expressive, appearance-related goods among women compared to men (Christenson et al., 1994, Dittmar et al., 1995).

Arousal seeking represents a further relevant component within this construct. Arousal is defined as a physiological state of increased alertness or excitement (Workman & Paper, 2010), while arousal seeking describes a dispositional tendency to seek intense or stimulating experiences, often involving risk (Zuckerman, 1994). In CBSD, arousal and pleasure have been shown to mediate buying-shopping decisions, particularly in younger cohorts (Ngo et al., 2024). Experimental findings further indicate that arousal responses are domain-specific rather than generalized: both hedonic (e.g., apparel) and utilitarian (e.g., grocery) buying-shopping contexts elicit elevated arousal, potentially reflecting affective excitement or internal self-control during purchase anticipation (Serfas et al., 2014). Accordingly, arousal may function as a physiological link between personality traits such as impulsivity and buying–shopping behavior. Consistent with this interpretation, CBSD has been repeatedly associated with heightened arousal-seeking tendencies (Workman & Paper, 2010), which are reflected in the proposed diagnostic criteria as “chase positive mood (e.g., pleasure, excitement, “high” while buying/shopping)” (Müller, Laskowski, et al., 2021).

In contrast, approach-oriented behaviors in BPD —particularly attention seeking— have primarily been conceptualized as manifestations of attachment dysregulation rather than reward- or arousal-driven approach motivation. Clinical accounts suggest that such behaviors serve to alleviate distress related to intolerance of aloneness and insecure attachment (Gunderson, 1996). Similarly, self-injurious behaviors in BPD are predominantly understood as strategies for distress or attachment regulation, rather than expressions of arousal- or reward-seeking traits (Naguy, 2025). Thus, despite superficial behavioral similarities, approach-oriented behaviors in CBSD and BPD appear to reflect qualitatively different underlying motivational mechanisms.

### 3.3 Constructs Predominantly Associated With Borderline Personality Disorder

The constructs with stronger empirical support for BPD are shown on the right-hand side of Fig. 1. This does not imply absence in CBSD but rather reflects that the available evidence is currently more consistent for BPD. Where evidence for CBSD is limited, indirect, or absent, this is explicitly stated and should not be interpreted as evidence of non-relevance.

#### 3.3.1 Internal Representations of Self and Others/Object Relations

Object relations refer to the internal mental representations of others that are shaped primarily by early interpersonal experiences and are central to the development of the self-concept. Mature object relations involve the capacity to recognize the uniqueness of relationships and to understand the nature of one’s connections with others (Blatt et al., 1997).

Although disturbances in object relations are not an explicit diagnostic criterion for BPD in either the DSM-5 (American Psychiatric Association, 2013) or the ICD-11 (World Health Organization, 2022), empirical evidence indicates that object relational functioning is highly relevant to the disorder. Cheek et al. (2021) demonstrated that poorer quality of object relations is a significant predictor of BPD severity and is uniquely associated with core BPD symptoms. Low-quality object relations are linked to the use of primitive defense mechanisms, controlling interpersonal behaviors, heightened sensitivity to perceived rejection or abandonment, a fragile self-image, and intense emotional responses to relational separation (Cheek et al., 2021).

To date, object relations or internal representations of self and others have not been systematically examined in the context of CBSD, and empirical evidence supporting the relevance of this construct for CBSD is currently lacking.

#### 3.3.2 Mentalization

Mentalization refers to the largely preconscious and imaginative capacity to understand and interpret human behavior in terms of underlying mental states such as thoughts, feelings, desires, and intentions (Fonagy et al., 2007).

Although deficits in mentalization are not explicit diagnostic criteria for BPD in either the DSM-5 (American Psychiatric Association, 2013) or the ICD-11 (World Health Organization, 2022), impaired and fragile mentalization is considered a core characteristic of the disorder (Bateman & Fonagy, 2010). Individuals with BPD often struggle to differentiate between their own mental states and those of others, which contributes to confusion in internal experience and interpersonal relationships and manifests in emotional instability and dysfunctional relational patterns (Fonagy et al., 2011). These difficulties are closely linked to attachment disturbances and identity diffusion, both central characteristics of BPD. Mentalization capacity is also highly sensitive to social and interpersonal stress, and individuals with BPD show significantly increased mentalization errors in both affective and cognitive dimensions (Bateman and Fonagy, 2010, Petersen et al., 2016).

To date, mentalization deficits have not been systematically examined in CBSD, and empirical evidence for impairments in mentalization within the CBSD literature is currently lacking.

#### 3.3.3 Chronic Emptiness

The inherently subjective nature of emptiness, defined by the absence of something, makes it particularly difficult to conceptualize with precision (Price et al., 2022). Its assessment is further complicated by the fact that individuals with BPD often struggle to consistently and coherently articulate their experience of emptiness (Miller et al., 2020). Subjective emptiness is characterized by profound feelings of disconnection from oneself and others, persistent unfulfillment, and a perceived lack of meaning, and is closely linked to internalizing symptoms such as negative emotions, interpersonal withdrawal, self-destructive behavior, and difficulties with identity (Price et al., 2022).

Chronic emptiness is a diagnostic criterion for BPD in both the DSM-5 (American Psychiatric Association, 2013) and the ICD-11 (World Health Organization, 2022) and is significantly correlated with core features of the disorder, including identity disturbance, dysphoria, interpersonal dysfunction, dissociation, self-harm, and suicidality (Elsner et al., 2018). Emptiness has also been identified as a predictor of suicide urges in individuals with BPD (Fulham et al., 2023) and, when experienced frequently and intensely, is associated with lower remission rates (Miller et al., 2020).

In contrast, evidence linking chronic emptiness to CBSD is sparse and indirect. Available findings suggest that emptiness-like experiences may mediate the relationship between narcissistic vulnerability and materialistic behavior (Reeves et al., 2012, Zerach, 2016). However, chronic emptiness as a persistent and pervasive experiential state comparable to that observed in BPD has not been systematically examined in CBSD.

#### 3.3.4 Negative Perceptual/Interpretation Bias

Negative interpretation bias refers to the tendency to perceive unclear or ambiguous situations as harmful or unfavorable (Sherman & Ehrenreich-May, 2017).

Although negative perceptual bias is not a specific diagnostic criterion for BPD in either the DSM-5 (American Psychiatric Association, 2013) or the ICD-11 (World Health Organization, 2022), individuals with BPD frequently misinterpret others’ emotional states and are more likely to perceive neutral or ambiguous facial expressions as negative (Mitchell et al., 2014). They may misread emotional expressions (Daros et al., 2014) and tend to evaluate positive or neutral self-relevant information as less positive (Winter et al., 2015). Such biases can result in responses that appear confusing or inappropriate, particularly in reaction to subtle or ambiguous facial expressions (Daros et al., 2014). Moreover, individuals with BPD show pronounced difficulties in recognizing and appropriately processing positive social signals, often exhibiting low baseline expectations of social acceptance and adjusting these expectations primarily in response to negative, but not positive feedback (Liebke et al., 2018).

Importantly, this bias appears to arise less from heightened reactivity to negative stimuli than from an absence of a positive interpretive bias when evaluating self-relevant positive or neutral stimuli (Winter et al., 2015). Trauma-related factors may further exacerbate these difficulties, as specific forms of childhood trauma, particularly sexual abuse, have been linked to impaired interpretation of neutral facial expressions (Pfaltz et al., 2019).

In contrast, negative perceptual or interpretation biases have not been systematically examined in CBSD. To date, there is no empirical evidence indicating that biased interpretation of ambiguous social or emotional information constitutes a core or defining feature of CBSD.

#### 3.3.5 Non-Suicidal Self-Injurious Behavior

NSSI is defined as the repeated act of causing physical harm to oneself without suicidal intent (American Psychiatric Association, 2013). In the DSM-5 (American Psychiatric Association, 2013), NSSI is listed in combination with recurrent suicidal behavior or ideation as a diagnostic criterion for BPD, whereas in the ICD-11 (World Health Organization, 2022) it is described as a stand-alone feature associated with the disorder.

NSSI and BPD frequently co-occur (Brickman et al., 2014). Common forms include cutting, banging the head against hard surfaces, burning, scalding, or cauterizing the skin, as well as high-risk behaviors such as reckless driving, which may also be conceptualized as self-harm (Bohus, 2019). These behaviors often begin with lower severity and escalate over time.

Difficulties in emotion regulation play a central role in the emergence and maintenance of NSSI, which functions as a maladaptive coping strategy in BPD (Bohus, 2019). Certain NSSI patterns have been shown to differentially predict BPD in adolescents (Stead et al., 2019). Moreover, impulsivity, chronic emptiness, and identity disturbance–core features of BPD–are each positively associated with a lifetime history of NSSI (Brickman et al., 2014).

In contrast, the relationship between NSSI and CBSD remains poorly understood. One study reported a positive association between NSSI and compulsive buying in women, but not in men (Raemen et al., 2020). Given the lack of replication and theoretical integration, current evidence does not support NSSI as a clinically meaningful or mechanistically shared feature of CBSD.

#### 3.3.6 Unstable Social Relationships

Relationship instability has been conceptualized as a dynamic and evolving process characterized by marked fluctuations in relational dynamics, largely driven by changes in commitment (Monk et al., 2025). Empirical evidence consistently indicates that unstable interpersonal relationships constitute a core feature of BPD. Women recently diagnosed with BPD reported insecurity, fear, and a lack of safety in close relationships, often relying on others or external objects for emotional security (Moltu et al., 2023).

Longitudinal data further demonstrate that BPD symptoms in adolescent girls are associated with a higher number of romantic relationships, heightened relational preoccupation, relational insecurity, and a strong drive to maintain relationships (Lazarus et al., 2019). These patterns increase the likelihood of emotionally intense, conflictual, and at times aggressive romantic relationships, which may, in turn, exacerbate BPD symptoms over time. Consistent with these findings, individuals with BPD commonly report dysfunctional romantic relationships characterized by insecure attachment, impaired communication, reduced relationship satisfaction, and frequent misinterpretation of partners’ behaviors, alongside verbal or physical aggression (Ociskova et al., 2023).

Unstable relationships in BPD are closely linked to identity disturbance, defined as a markedly and persistently unstable self-image or sense of self (American Psychiatric Association, 2013, World Health Organization, 2022). Individuals with BPD tend to interpret negative and neutral social events more negatively and show selective memory biases toward self-referential social information, reflecting disrupted self-referential processing (Winter et al., 2016). Expectations of devaluation and rejection further contribute to interpersonal difficulties (Bohus et al., 2021). Even in objectively inclusive social situations, individuals with BPD may perceive exclusion and respond with uncooperative behavior (Liebke et al., 2018). Additional challenges include difficulties regulating interpersonal closeness and distance; pronounced fear of abandonment, combined with limited object permanence, often leads to misinterpretation of temporary separation as abandonment (Bohus, 2019).

In contrast, while CBSD has been associated with reduced social satisfaction, loneliness, and impaired psychosocial functioning, there is currently no systematic evidence that relationship instability constitutes a dynamic, self-perpetuating interpersonal pattern comparable to that observed in BPD.

#### 3.3.7 Dissociative Symptoms

Dissociation is a transdiagnostic phenomenon encompassing a range of symptoms characterized by temporary disruption in the perception of time, space, and the sense of self (American Psychiatric Association, 2013, Bohus, 2019).

Stress-related dissociation is a core feature of BPD in both the DSM-5 (American Psychiatric Association, 2013) and ICD-11 (World Health Organization, 2022). Dissociation occurs more frequently–though not exclusively–in individuals with BPD who have a history of traumatic experiences (Bohus et al., 2021). Recent findings indicate that dissociation in BPD may be particularly pronounced in contexts involving intimacy and vulnerability. For example, individuals with BPD report elevated dissociative symptoms in sexual situations compared to individuals without psychiatric diagnoses, possibly reflecting trauma-related processes and difficulties in affect regulation during close interpersonal interactions (Mazinan et al., 2024). Furthermore, dissociative symptom severity appears relatively stable across developmental stages, as no significant differences have been observed between adolescents and adults with BPD (Zanarini et al., 2023), supporting the notion of dissociation as a persistent feature of the disorder.

In contrast, although dissociation-related processes have recently been discussed in the context of CBSD, the available evidence suggests a qualitatively different role. Dissociation has been shown to mediate the relationship between alexithymia and compulsive buying, potentially functioning as a situational coping mechanism to manage emotional overload (Gori et al., 2024). Importantly, dissociative symptoms in CBSD appear to be state-dependent and context-specific rather than stress-related and pervasive. Thus, while dissociation may contribute to compulsive buying as a transient affect-regulatory strategy, it does not constitute a defining or diagnostically central feature comparable to dissociation in BPD.

## 4 Discussion

The observed comorbidity between CBSD and BPD is most plausibly explained by overlapping etiological risk factors, as illustrated in Fig. 1 and Table 1. Both disorders are associated with shared, largely unspecific vulnerability factors, including traumatic experiences (David et al., 2024, Laskowski et al., 2019, Sansone et al., 2013), elevated levels of general psychopathology, and reduced quality of life (IsHak et al., 2013, Zhang et al., 2017).

The constructs included in the proposed framework were allocated to CBSD or BPD based on the relative strength, consistency, and specificity of the available empirical evidence. Importantly, this allocation does not imply exclusivity. Rather, constructs categorized as predominantly associated with one disorder may also be relevant to the other. A central limitation of this approach is that apparent disorder-specificity may reflect true clinical distinctions but may equally result from uneven research attention across disorders. In this context, the absence of evidence must not be conflated with evidence of absence, particularly given the inherent limitations of null findings from a test-theoretical perspective (Cohen, 1994). Consistent with this, several constructs initially assumed to be disorder-specific have since demonstrated relevance across both CBSD and BPD.

Many of the constructs discussed in this framework are likely interrelated. However, the predominance of cross-sectional study designs precludes conclusions regarding directionality or causality. For example, decision-making deficits may arise because of heightened negative affect (e.g., anxiety or depressive symptoms), but could also be driven by maladaptive cognitive styles, such as perfectionism or intolerance of uncertainty (Kyrios et al., 2004). Longitudinal and experimental designs are therefore essential to disentangle these relationships.

A major limitation of the present work is the absence of a formal systematic literature search. Although the synthesis was informed by extensive expertise and iterative cross-checking, the construct allocation relied on selected sources and expert consensus and may have overlooked relevant studies. Additionally, most empirical findings were derived from cross-sectional data, further limiting causal inference. Variability in construct definitions and operationalizations across studies also complicates direct comparison and integration of results.

Future research should aim to systematically and explicitly examine the identified constructs across both CBSD and BPD populations. Investigating constructs currently considered atypical for one disorder within the other may help clarify genuine overlaps versus disorder-specific mechanisms. Such work would facilitate a more precise differentiation between shared and unique processes and support the development of both transdiagnostic and disorder-specific interventions.

A more fine-grained understanding of the cognitive–affective mechanisms underlying CBSD and BPD is critical for refining diagnostic models, improving treatment planning, and identifying clinically meaningful intervention targets. For example, craving has been robustly linked to relapse risk in behavioral addictions and may represent a particularly relevant treatment focus in CBSD. Integrating craving-related mechanisms into diagnostic and therapeutic frameworks could enhance both classification consistency and treatment effectiveness. More broadly, acknowledging the complex interplay between impulsivity, identity-related vulnerabilities, and emotional dysregulation may contribute to earlier identification and more personalized interventions in both disorders.

Crucially, the present framework does not assume equivalence between CBSD and BPD across domains such as identity disturbance, emotion regulation, or inhibitory control. Instead, it highlights differences in depth, stability, and clinical significance. The constructs are therefore compared at a functional level rather than in terms of diagnostic severity or structural personality pathology. The value of juxtaposing CBSD and BPD does not lie in suggesting global similarity, but in demonstrating partial convergence in emotion-driven impulsivity, self-referential regulation, and identity-related vulnerabilities, alongside marked divergence in structural personality pathology, interpersonal dysfunction, and affective instability.

Declarations

## CRediT authorship contribution statement

**Nora M. Laskowski:** Writing – review & editing, Writing – original draft, Visualization, Project administration, Methodology, Investigation, Data curation, Conceptualization. **Luisa Sabel:** Writing – review & editing, Writing – original draft, Methodology, Investigation, Data curation. **Gerrit Brandt:** Writing – review & editing, Writing – original draft, Methodology, Investigation, Data curation. **Georgios Paslakis:** Writing – review & editing, Validation, Supervision.

## Ethics approval and consent to participate

Not applicable

Consent for publication

Not applicable

Availability of data and materials

Not applicable

## Funding

Not applicable

## Declaration of competing interest

The authors declare that they have no known competing financial interests or personal relationships that could have appeared to influence the work reported in this paper.

## Data Availability

No data was used for the research described in the article.

## Footnotes

1 Due to the lack of an official recognition as a distinct mental disorder, a wide range of terms has been used to describe pathological buying/shopping behavior. For consistency with the ICD-11 framework, we use the term Compulsive Buying-Shopping Disorder (CBSD) throughout this work, while retaining the original terminology used in individual studies when referring to specific findings.
