# Supplementary material for: Overlapping Phenotypes of Compulsive Buying-Shopping Disorder and Borderline Personality Disorder: An Evidence-Based Model
Source: Addict Behav Rep. 2026 Jan 23;23:100669. doi: 10.1016/j.abrep.2026.100669 (PMC12874595; doi:10.1016/j.abrep.2026.100669)
Supplement: Supplementary Data 1 [file mmc1.docx]

**Overlapping Phenotypes of Compulsive Buying-Shopping Disorder and Borderline Personality Disorder: An Evidence-Based Model – Supplementary Material**

Supplementary Table 1: Overlapping features of Compulsive Buying-Shopping Disorder and Borderline Personality Disorder

| **Reference** | **Construct (Result Section)** |
| --- | --- |
| Claes & Müller, 2017; David et al., 2024; Kyrios et al., 2004; Nica & Links, 2009; Selby et al., 2009; Soloff et al., 2017 | Affective Regulation and Emotional Stability |
| Christenson et al., 1994; Dittmar et al., 1995; Gunderson, 1996; Hanley & Wilhelm, 1992; Kellett & Bolton, 2009; Müller, Claes, et al., 2021; Naguy, 2025; Ngo et al., 2024; Serfas et al., 2014; Workman & Paper, 2010; Zuckerman, 1994 | Approach-Oriented Personality Traits |
| Etxaburu et al., 2024; Gao et al., 2017; Hashworth et al., 2021; Norberg et al., 2018; Smith & South, 2020; Topino et al., 2022 | Attachment Style and Emotional Dependence |
| Cardasis et al., 1997; David & Norberg, 2022; Dittmar, 2005b; Dittmar et al., 1995; Frost et al., 2007; Kyrios et al., 2004; Moulding et al., 2017; Müller, Claes, et al., 2021; Scherhorn et al., 1990; Timpano & Port, 2021 | Attachment to Possessions |
| Berenson et al., 2021; Bijttebier et al., 2009; Bilge & Emiral, 2022; Claes et al., 2011; Claes & Müller, 2017; Ross et al., 2013; Verplanken & Sato, 2011; Voth et al., 2014 | Behavioral Inhibition and Behavioral Activation System |
| Elsner et al., 2018; Fulham et al., 2023; Miller et al., 2020; Reeves et al., 2012; Zerach, 2016 | Chronic Emptiness |
| Brand et al., 2019, 2025; Christenson et al., 1994; Frost et al., 2002; Kellett & Bolton, 2009; Lejoyeux et al., 2005; Lutz et al., 2022; Müller et al., 2007; Theisejans et al., 2025; Varvaras et al., 2025 | Compulsivity |
| Elango et al., 2025; Trotzke et al., 2014, 2019; Victor et al., 2012 | Craving and Cue-Reactivity |
| Bajzát et al., 2023; Bechara et al., 1994; Derbyshire et al., 2014; Kyrios et al., 2004; Lo & Harvey, 2012; Nicolai & Moshagen, 2017; Paret et al., 2017; Trotzke et al., 2015 | Decision-Making |
| Gratz & Roemer, 2004; Gross, 1998, 2015 | Definition Affective Regulation and Emotional Stability |
| Elliot & Thrash, 2002; Mehrabian & Russell, 1973; Mellor, 2005 | Definition Approach-Oriented Personality Traits |
| Bartholomew & Horowitz, 1991 | Definition Attachment Style and Emotional Dependence |
| Ball & Tasaki, 1992 | Definition Attachment to Possessions |
| Gray, 1970, 1982 | Definition Behavioral Inhibition and Behavioral Activation System |
| Miller et al., 2020; Price et al., 2022 | Definition Chronic Emptiness |
| Fineberg et al., 2014 | Definition Compulsivity |
| Tiffany & Wray, 2012 | Definition Craving and Cue-Reactivity |
| Harris, 1998; Jones et al., 2019 | Definition Decision-Making |
| American Psychiatric Association, 2013; Bohus, 2019 | Definition Dissociative Symptoms |
| Evenden, 1999 | Definition Impulsivity |
| Blatt et al., 1997 | Definition Internal Representations of Self and Others/Object Relations |
| Kellett & Bolton, 2009 | Definition Materialism |
| Fonagy et al., 2007 | Definition Mentalization |
| Sherman & Ehrenreich-May, 2017 | Definition Negative Perceptual/Interpretation Bias |
| American Psychiatric Association, 2013; World Health Organization, 2022 | Definition Non-Suicidal Self-Injurious Behavior |
| Cloninger et al., 1994 | Definition Novelty Seeking |
| Hewitt & Flett, 1991 | Definition Perfectionism |
| Guenther et al., 2016 | Definition Self-Concept and Identity |
| Baumeister & Vohs, 2004 | Definition Self-Regulation and -Control |
| Monk et al., 2025 | Definition Unstable Social Relationships |
| American Psychiatric Association, 2013; World Health Organization, 2022 | Diagnostic Criteria Borderline Personality Disorder |
| Laskowski et al., 2021; Müller, Laskowski, et al., 2021 | Diagnostic Criteria Compulsive Buying-Shopping Disorder |
| Bohus et al., 2021; Gori et al., 2024; Mazinan et al., 2024; Zanarini et al., 2010 | Dissociative Symptoms |
| Billieux et al., 2008; Black et al., 2012; Davenport et al., 2012; Sebastian et al., 2013; Soloff et al., 2017 | Impulsivity |
| Cheek et al., 2021 | Internal Representations of Self and Others/Object Relations |
| Claes et al., 2016; Müller et al., 2020, 2022; Villardefrancos & Otero-López, 2016; Wegmann et al., 2023 | Materialism |
| Bateman & Fonagy, 2010; Fonagy et al., 2011; Petersen et al., 2016 | Mentalization |
| Daros et al., 2014; Liebke et al., 2018; Mitchell et al., 2014; Pfaltz et al., 2019; Winter et al., 2015 | Negative Perceptual/Interpretation Bias |
| Bohus, 2019; Brickman et al., 2014; Raemen et al., 2020; Stead et al., 2019 | Non-Suicidal Self-Injurious Behavior |
| Barnow et al., 2007; Black et al., 2012; Bozzatello et al., 2024; Claes & Müller, 2017; Di Nicola et al., 2010; Fernández-Aranda et al., 2019; Fossati et al., 2001; Jiménez-Murcia et al., 2015; Joyce et al., 2003; Kaess et al., 2013; Machado et al., 2022 | Novelty Seeking |
| Chen et al., 2019; Dimaggio et al., 2018; Kyrios et al., 2004; Martinelli et al., 2014; Stoeber, 2014 | Perfectionism |
| Claes et al., 2016; Dittmar, 2005a; Faggioli et al., 2024; Müller et al., 2020; Noguti & Bokeyar, 2014; Sharif & Khanekharab, 2017; Wilkinson-Ryan & Westen, 2000 | Self-Concept and Identity |
| Billieux et al., 2008, 2010; Bud et al., 2023; Euler et al., 2021; Johnson et al., 2017; King-Casas et al., 2008; Lindheimer et al., 2020; Lozano et al., 2016; Van Malderen et al., 2024; Vogt et al., 2015 | Self-Regulation and -Control |
| Bohus et al., 2021; Lazarus et al., 2019; Liebke et al., 2018; Moltu et al., 2023; Ociskova et al., 2023; Winter et al., 2016 | Unstable Social Relationships |

**References**

American Psychiatric Association. (2013). *DSM-5 Table of Contents*. American Psychiatric Association. www.DSM5.org

Bajzát, B., Soltész, P., Soltész-Várhelyi, K., Lévay, E. E., & Unoka, Z. S. (2023). Impaired decision-making in borderline personality disorder. *Frontiers in Psychology*, *14*. https://doi.org/10.3389/fpsyg.2023.1109238

Ball, A. D., & Tasaki, L. H. (1992). The Role and Measurement of Attachment in Consumer Behavior. *The Role and Measurement of Attachment in Consumer Behavior*, *1*(2), 155–172.

Barnow, S., Herpertz, S. C., Spitzer, C., Stopsack, M., Preuss, U. W., Grabe, H. J., Kessler, C., & Freyberger, H. J. (2007). Temperament and Character in Patients with Borderline Personality Disorder Taking Gender and Comorbidity into Account. *Psychopathology*, *40*(6), 369–378. https://doi.org/10.1159/000106467

Bartholomew, K., & Horowitz, L. M. (1991). Attachment Styles Among Young Adults :A Test of a Four-Category Model. *Journal of Personality and Social Psychology*, *2*, 226–244.

Bateman, A., & Fonagy, P. (2010). Mentalization based treatment for borderline personality disorder. *World Psychiatry*, *9*, 11–15.

Baumeister, R. F., & Vohs, K. D. (2004). *Handbook of self-regulation: Research, theory, and applications*. Guilford Press.

Bechara, A., Damasio, A. R., Damasio, H., & Anderson, S. W. (1994). Insensitivity to future consequences following damage to human prefrontal cortex. *Cognition*, *50*(1–3), 7–15. https://doi.org/10.1016/0010-0277(94)90018-3

Berenson, K. R., Van De Weert, S. M., Nicolaou, S., Campoverde, C., Rafaeli, E., & Downey, G. (2021). Reward and Punishment Sensitivity in Borderline and Avoidant Personality Disorders. *Journal of Personality Disorders*, *35*(4), 573–588. https://doi.org/10.1521/pedi_2020_34_475

Bijttebier, P., Beck, I., Claes, L., & Vandereycken, W. (2009). Gray’s Reinforcement Sensitivity Theory as a framework for research on personality–psychopathology associations. *Clinical Psychology Review*, *29*(5), 421–430. https://doi.org/10.1016/j.cpr.2009.04.002

Bilge, Y., & Emiral, E. (2022). The mediator role of BIS/BAS Systems in the Relationship between psychological symptoms and borderline personality features: Conformation from a non-Western sample. *Current Psychology*, *41*(12), 9008–9018. https://doi.org/10.1007/s12144-021-01386-3

Billieux, J., Gay, P., Rochat, L., & Van der Linden, M. (2010). The role of urgency and its underlying psychological mechanisms in problematic behaviours. *Behaviour Research and Therapy*, *48*(11), 1085–1096. https://doi.org/10.1016/j.brat.2010.07.008

Billieux, J., Rochat, L., Rebetez, M. M. L., & Van der Linden, M. (2008). Are all facets of impulsivity related to self-reported compulsive buying behavior? *Personality and Individual Differences*, *44*(6), 1432–1442. https://doi.org/10.1016/j.paid.2007.12.011

Black, D. W., Shaw, M., McCormick, B., Bayless, J. D., & Allen, J. (2012). Neuropsychological performance, impulsivity, ADHD symptoms, and novelty seeking in compulsive buying disorder. *Psychiatry Research*, *200*(2–3), 581–587. https://doi.org/10.1016/j.psychres.2012.06.003

Blatt, S. J., Auerbach, J. S., & Levy, K. N. (1997). Mental Representations in Personality Development, Psychopathology, and the Therapeutic Process. *Review of General Psychology*, *1*(4), 351–374. https://doi.org/10.1037/1089-2680.1.4.351

Bohus, M. (2019). *Borderline-Störung: Vol. 2<*. Hogrefe Verlag GmbH & Co. KG.

Bohus, M., Stoffers-Winterling, J., Sharp, C., Krause-Utz, A., Schmahl, C., & Lieb, K. (2021). Borderline personality disorder. *The Lancet*, *398*(10310), 1528–1540. https://doi.org/10.1016/S0140-6736(21)00476-1

Bozzatello, P., Blua, C., Brandellero, D., Baldassarri, L., Brasso, C., Rocca, P., & Bellino, S. (2024). Gender differences in borderline personality disorder: a narrative review. *Frontiers in Psychiatry*, *15*. https://doi.org/10.3389/fpsyt.2024.1320546

Brand, M., Müller, A., Wegmann, E., Antons, S., Brandtner, A., Müller, S. M., Stark, R., Steins-Loeber, S., & Potenza, M. N. (2025). Current interpretations of the I-PACE model of behavioral addictions. *Journal of Behavioral Addictions*. https://doi.org/10.1556/2006.2025.00020

Brand, M., Wegmann, E., Stark, R., Müller, A., Wölfling, K., Robbins, T. W., & Potenza, M. N. (2019). The Interaction of Person-Affect-Cognition-Execution (I-PACE) model for addictive behaviors: Update, generalization to addictive behaviors beyond internet-use disorders, and specification of the process character of addictive behaviors. *Neuroscience and Biobehavioral Reviews*, *104*, 1–10. https://doi.org/10.1016/j.neubiorev.2019.06.032

Brickman, L. J., Ammerman, B. A., Look, A. E., Berman, M. E., & McCloskey, M. S. (2014). The relationship between non-suicidal self-injury and borderline personality disorder symptoms in a college sample. *Borderline Personality Disorder and Emotion Dysregulation*, *1*(1). https://doi.org/10.1186/2051-6673-1-14

Bud, S., Nechita, D., & Szentagotai Tatar, A. (2023). Emotion regulation strategies in borderline personality disorder: a meta-analysis. *Clinical Psychologist*, *27*(2), 142–159. https://doi.org/10.1080/13284207.2022.2152668

Cardasis, W., Hochman, J. A., & Silk, K. R. (1997). Transitional Objects and Borderline Personality Disorder. *Am J Psychiatry*, *154*(2).

Cheek, J., Kealy, D., Joyce, A., & Ogrodniczuk, J. (2021). Borderline personality disorder as a syndrome of poor quality of object relations. *Archives of Psychiatry and Psychotherapy*, *23*(2), 7–14. https://doi.org/10.12740/APP/128104

Chen, C., Hewitt, P. L., Flett, G. L., & Roxborough, H. M. (2019). Multidimensional perfectionism and borderline personality organization in emerging adults: A two-wave longitudinal study. *Personality and Individual Differences*, *146*, 143–148. https://doi.org/10.1016/j.paid.2019.04.011

Christenson, G. A., Faber, R. J., de Zwaan, M., Raymond, N. C., Specker, S. M., Ekern, M. D., Mackenzie, T. B., Crosby, R. D., Crow, S. J., & Eckert, E. D. (1994). Compulsive buying: descriptive characteristics and psychiatric comorbidity. *The Journal of Clinical Psychiatry*, *55*(1), 5–11.

Claes, L., Bijttebier, P., Mitchell, J. E., De Zwaan, M., & Mueller, A. (2011). The relationship between compulsive buying, eating disorder symptoms, and temperament in a sample of female students. *Comprehensive Psychiatry*, *52*(1), 50–55. https://doi.org/10.1016/j.comppsych.2010.05.003

Claes, L., & Müller, A. (2017). Resisting temptation: Is compulsive buying an expression of personality deficits? *Current Addiction Reports*, *4*, 237–245. https://doi.org/10.1007/s40429-017-0152-0

Claes, L., Müller, A., & Luyckx, K. (2016). Compulsive buying and hoarding as identity substitutes: The role of materialistic value endorsement and depression. *Comprehensive Psychiatry*, *68*, 65–71. https://doi.org/10.1016/j.comppsych.2016.04.005

Cloninger, C. R., Przybeck, T. R., Svrakic, D. M., & Wetzel, R. D. (1994). *The Temperament and Character Inventory (TCI): A guide to its development and use.* .

Daros, A. R., Uliaszek, A. A., & Ruocco, A. C. (2014). Perceptual biases in facial emotion recognition in borderline personality disorder. *Personality Disorders: Theory, Research, and Treatment*, *5*(1), 79–87. https://doi.org/10.1037/per0000056

Davenport, K., Houston, J. E., & Griffiths, M. D. (2012). Excessive Eating and Compulsive Buying Behaviours in Women: An Empirical Pilot Study Examining Reward Sensitivity, Anxiety, Impulsivity, Self-Esteem and Social Desirability. *International Journal of Mental Health and Addiction*, *10*(4), 474–489. https://doi.org/10.1007/s11469-011-9332-7

David, J., Kim, H. S., Hodgins, D. C., Dawson, S. J., Tabri, N., Shead, N. W., & Keough, M. T. (2024). Emotional difficulties mediate the impact of adverse childhood experiences on compulsive buying-shopping problems. *Journal of Behavioral Addictions*. https://doi.org/10.1556/2006.2024.00056

David, J., & Norberg, M. M. (2022). Redefining object attachment: Development and validation of a new scale. *Journal of Behavioral Addictions*, *11*(3), 941–951. https://doi.org/10.1556/2006.2022.00058

Derbyshire, K. L., Chamberlain, S. R., Odlaug, B. L., Schreiber, L. R. N., & Grant, J. E. (2014). Neurocognitive functioning in compulsive buying disorder. *Annals of Clinical Psychiatry*, *26*(1), 57–63.

Di Nicola, M., Tedeschi, D., Mazza, M., Martinotti, G., Harnic, D., Catalano, V., Bruschi, A., Pozzi, G., Bria, P., & Janiri, L. (2010). Behavioural addictions in bipolar disorder patients: Role of impulsivity and personality dimensions. *Journal of Affective Disorders*, *125*(1–3), 82–88. https://doi.org/10.1016/j.jad.2009.12.016

Dimaggio, G., MacBeth, A., Popolo, R., Salvatore, G., Perrini, F., Raouna, A., Osam, C. S., Buonocore, L., Bandiera, A., & Montano, A. (2018). The problem of overcontrol: Perfectionism, emotional inhibition, and personality disorders. *Comprehensive Psychiatry*, *83*, 71–78. https://doi.org/10.1016/j.comppsych.2018.03.005

Dittmar, H. (2005a). A New Look at “Compulsive Buying”: Self–Discrepancies and Materialistic Values as Predictors of Compulsive Buying Tendency. *Journal of Social and Clinical Psychology*, *24*(6), 832–859. https://doi.org/10.1521/jscp.2005.24.6.832

Dittmar, H. (2005b). Compulsive buying - A growing concern? An examination of gender, age, and endorsement of materialistic values as predictors. *British Journal of Psychology*, *96*(4), 467–491. https://doi.org/10.1348/000712605X53533

Dittmar, H., Beattie, J., & Friese, S. (1995). Gender identity and material symbols: Objects and decision considerations in impulse purchases. *Journal of Economic Psychology*, *16*, 491–511.

Elango, S. C., Sharma, E., & Roopesh, B. N. (2025). Impulsive-addictive-compulsive Types of Non-suicidal Self-injury: A Case Series. *Indian Journal of Psychological Medicine*. https://doi.org/10.1177/02537176241300760

Elliot, A. J., & Thrash, T. M. (2002). Approach-avoidance motivation in personality: Approach and avoidance temperaments and goals. *Journal of Personality and Social Psychology*, *82*(5), 804–818. https://doi.org/10.1037/0022-3514.82.5.804

Elsner, D., Broadbear, J. H., & Rao, S. (2018). What is the clinical significance of chronic emptiness in borderline personality disorder? *Australasian Psychiatry*, *26*(1), 88–91. https://doi.org/10.1177/1039856217734674

Etxaburu, N., Momeñe, J., Herrero, M., Chávez-Vera, M. D., Olave, L., Iruarrizaga, I., & Estévez, A. (2024). Buying-shopping disorder, impulsivity, emotional dependence and attachment in adolescents. *Current Psychology*, *43*(2), 1507–1518. https://doi.org/10.1007/s12144-023-04425-3

Euler, S., Nolte, T., Constantinou, M., Griem, J., Montague, P. R., & Fonagy, P. (2021). Interpersonal Problems in Borderline Personality Disorder: Associations With Mentalizing, Emotion Regulation, and Impulsiveness. *Journal of Personality Disorders*, *35*(2), 177–193. https://doi.org/10.1521/pedi_2019_33_427

Evenden, J. L. (1999). Varieties of impulsivity. *Psychopharmacology*, *146*, 348–361. https://doi.org/10.1007/PL00005481

Faggioli, I., Esposito, C. M., & Stanghellini, G. (2024). Identity and Temporal Fragmentation in Borderline Personality Disorder: A Systematic Review. *Brain Sciences*, *14*(12), 1221. https://doi.org/10.3390/brainsci14121221

Fernández-Aranda, F., Granero, R., Mestre-Bach, G., Steward, T., Müller, A., Brand, M., Mena-Moreno, T., Vintró-Alcaraz, C., Del Pino-Gutiérrez, A., Moragas, L., Mallorquí-Bagué, N., Aymamí, N., Gómez-Peña, M., Lozano-Madrid, M., Menchón, J. M., & Jiménez-Murcia, S. (2019). Spanish validation of the pathological buying screener in patients with eating disorder and gambling disorder. *Journal of Behavioral Addictions*, *8*(1), 123–134. https://doi.org/10.1556/2006.8.2019.08

Fineberg, N. A., Chamberlain, S. R., Goudriaan, A. E., Stein, D. J., Vanderschuren, L. J. M. J., Gillan, C. M., Shekar, S., Gorwood, P. A. P. M., Voon, V., Morein-Zamir, S., Denys, D., Sahakian, B. J., Moeller, F. G., Robbins, T. W., & Potenza, M. N. (2014). New developments in human neurocognition: clinical, genetic, and brain imaging correlates of impulsivity and compulsivity. *CNS Spectrums*, *19*(1), 69–89. https://doi.org/10.1017/S1092852913000801

Fonagy, P., Gergely, G., & Target, M. (2007). The parent–infant dyad and the construction of the subjective self. *Journal of Child Psychology and Psychiatry*, *48*(3–4), 288–328. https://doi.org/10.1111/j.1469-7610.2007.01727.x

Fonagy, P., Luyten, P., & Strathearn, L. (2011). Borderline personality disorder, mentalization, and the neurobiology of attachment. *Infant Mental Health Journal*, *32*(1), 47–69. https://doi.org/10.1002/imhj.20283

Fossati, A., Donati, D., Donini, M., Novella, L., Bagnato, M., & Maffei, C. (2001). Temperament, Character, and Attachment Patterns in Borderline Personality Disorder. *Journal of Personality Disorders*, *15*(5), 390–402. https://doi.org/10.1521/pedi.15.5.390.19197

Frost, R. O., Kyrios, M., McCarthy, K. D., & Matthews, Y. (2007). Self-Ambivalence and Attachment to Possessions. *Journal of Cognitive Psychotherapy*, *21*(3), 232–242. https://doi.org/10.1891/088983907781494582

Frost, R. O., Steketee, G., & Williams, L. (2002). Compulsive Buying, Compulsive Hoarding, and Obsessive-Compulsive Disorder. *Behavior Therapy*, *33*(2), 201–214. https://doi.org/10.1016/S0005-7894(02)80025-9

Fulham, L., Forsythe, J., & Fitzpatrick, S. (2023). The relationship between emptiness and suicide and <scp>self‐injury</scp> urges in borderline personality disorder. *Suicide and Life-Threatening Behavior*, *53*(3), 362–371. https://doi.org/10.1111/sltb.12949

Gao, S., Assink, M., Cipriani, A., & Lin, K. (2017). Associations between rejection sensitivity and mental health outcomes: A meta-analytic review. In *Clinical Psychology Review* (Vol. 57, pp. 59–74). Elsevier Inc. https://doi.org/10.1016/j.cpr.2017.08.007

Gori, A., Topino, E., Fioravanti, G., & Casale, S. (2024). Exploring the Psychodynamics of Compulsive Shopping: Single and Moderated Mediation Analyses. *International Journal of Mental Health and Addiction*, *22*(4), 2149–2165. https://doi.org/10.1007/s11469-022-00977-w

Gratz, K. L., & Roemer, L. (2004). Multidimensional Assessment of Emotion Regulation and Dysregulation: Development, Factor Structure, and Initial Validation of the Difficulties in Emotion Regulation Scale 1. In *Journal of Psychopathology and Behavioral Assessment* (Vol. 26, Issue 1).

Gray, J. A. (1970). The psychophysiological basis of introversion-extraversion. *Behaviour Research and Therapy*, *8*(3), 249–266. https://doi.org/10.1016/0005-7967(70)90069-0

Gray, J. A. (1982). Précis of  The neuropsychology of anxiety: An enquiry into the functions of the septo-hippocampal system. *Behavioral and Brain Sciences*, *5*(3), 469–484. https://doi.org/10.1017/S0140525X00013066

Gross, J. J. (1998). Antecedent- and response-focused emotion regulation: Divergent consequences for experience, expression, and physiology. *Journal of Personality and Social Psychology*, *74*(1), 224–237. https://doi.org/10.1037/0022-3514.74.1.224

Gross, J. J. (2015). Emotion Regulation: Current Status and Future Prospects. *Psychological Inquiry*, *26*(1), 1–26. https://doi.org/10.1080/1047840X.2014.940781

Guenther, C., Wilton, E., & Fernandes, R. (2016). Identity. In *Encyclopedia of Personality and Individual Differences* (pp. 1–10). Springer International Publishing. https://doi.org/10.1007/978-3-319-28099-8_1132-1

Gunderson, J. G. (1996). The borderline patient’s intolerance of aloneness: insecure attachments and therapist availability. *American Journal of Psychiatry*, *153*(6), 752–758. https://doi.org/10.1176/ajp.153.6.752

Hanley, A., & Wilhelm, M. S. (1992). Compulsive buying: An exploration into self-esteem and money attitudes. *Journal of Economic Psychology*, *13*(1), 5–18. https://doi.org/10.1016/0167-4870(92)90049-D

Harris, R. (1998). *Introduction to Decision Making*. VirtualSalt. http://www.virtualsalt.com/crebook5.htm

Hashworth, T., Reis, S., & Grenyer, B. F. S. (2021). Personal Agency in Borderline Personality Disorder: The Impact of Adult Attachment Style. *Frontiers in Psychology*, *12*. https://doi.org/10.3389/fpsyg.2021.669512

Hewitt, P. L., & Flett, G. L. (1991). Perfectionism in the self and social contexts: Conceptualization, assessment, and association with psychopathology. *Journal of Personality and Social Psychology*, *60*(3), 456–470. https://doi.org/10.1037/0022-3514.60.3.456

Jiménez-Murcia, S., Granero, R., Moragas, L., Steiger, H., Israel, M., Aymamí, N., Gómez-Peña, M., Sauchelli, S., Agüera, Z., Sánchez, I., Riesco, N., Penelo, E., Menchón, J. M., & Fernández-Aranda, F. (2015). Differences and similarities between bulimia nervosa, compulsive buying and gambling disorder. *European Eating Disorders Review*, *23*(2), 126–132. https://doi.org/10.1002/erv.2340

Johnson, B. N., Ashe, M. L., & Wilson, S. J. (2017). Self-Control Capacity as a Predictor of Borderline Personality Disorder Features, Problematic Drinking, and Their Co-occurrence. *Journal of Personality Disorders*, *31*(3), 289–305. https://doi.org/10.1521/pedi_2016_30_249

Jones, K. A., Hewson, T., Sales, C. P., & Khalifa, N. (2019). A Systematic Review and Meta-Analysis of Decision-Making in Offender Populations with Mental Disorder. *Neuropsychology Review*, *29*(2), 244–258. https://doi.org/10.1007/s11065-018-09397-x

Joyce, P. R., Mckenzie, J. M., Luty, S. E., Mulder, R. T., Carter, J. D., Sullivan, P. F., & Robert Cloninger, C. (2003). Temperament, childhood environment and psychopathology as risk factors for avoidant and borderline personality disorders. *Australian & New Zealand Journal of Psychiatry*, *37*(6), 756–764. https://doi.org/10.1080/j.1440-1614.2003.01263.x

Kaess, M., Resch, F., Parzer, P., von Ceumern-Lindenstjerna, I.-A., Henze, R., & Brunner, R. (2013). Temperamental Patterns in Female Adolescents With Borderline Personality Disorder. *Journal of Nervous & Mental Disease*, *201*(2), 109–115. https://doi.org/10.1097/NMD.0b013e31827f6480

Kellett, S., & Bolton, J. V. (2009). Compulsive buying: A cognitive-Behavioural model. *Clinical Psychology and Psychotherapy*, *16*(2), 83–99. https://doi.org/10.1002/cpp.585

King-Casas, B., Sharp, C., Lomax-Bream, L., Lohrenz, T., Fonagy, P., & Montague, P. R. (2008). The Rupture and Repair of Cooperation in Borderline Personality Disorder. *Science*, *321*(5890), 806–810. https://doi.org/10.1126/science.1156902

Kyrios, M., Frost, R. O., & Steketee, G. (2004). Cognitions in Compulsive Buying and Acquisition. *Cognitive Therapy and Research*, *28*(2), 241–258. https://doi.org/10.1023/B:COTR.0000021543.62799.32

Laskowski, N. M., Trotzke, P., De Zwaan, M., Brand, M., & Müller, A. (2021). Deutsche Übersetzung der Diagnosekriterien für die Kauf-Shopping-Störung. *Sucht*, *67*(6), 323–330. https://doi.org/10.1024/0939-5911/a000737

Lazarus, S. A., Choukas-Bradley, S., Beeney, J. E., Byrd, A. L., Vine, V., & Stepp, S. D. (2019). Too Much Too Soon?: Borderline Personality Disorder Symptoms and Romantic Relationships in Adolescent Girls. *Journal of Abnormal Child Psychology*, *47*(12), 1995–2005. https://doi.org/10.1007/s10802-019-00570-1

Lejoyeux, M., Bailly, F., Moula, H., Loi, S., & Adès, J. (2005). Study of compulsive buying in patients presenting obsessive-compulsive disorder. *Comprehensive Psychiatry*, *46*(2), 105–110. https://doi.org/10.1016/j.comppsych.2004.07.027

Liebke, L., Koppe, G., Bungert, M., Thome, J., Hauschild, S., Defiebre, N., Izurieta Hidalgo, N. A., Schmahl, C., Bohus, M., & Lis, S. (2018). Difficulties with being socially accepted: An experimental study in borderline personality disorder. *Journal of Abnormal Psychology*, *127*(7), 670–682. https://doi.org/10.1037/abn0000373

Lindheimer, N., Nicolai, J., & Moshagen, M. (2020). General rather than specific: Cognitive deficits in suppressing task irrelevant stimuli are associated with buying-shopping disorder. *PLoS ONE*, *15*(8 August). https://doi.org/10.1371/journal.pone.0237093

Lo, H. Y., & Harvey, N. (2012). Effects of shopping addiction on consumer decision-making: Web-based studies in real time. *Journal of Behavioral Addictions*, *1*(4), 162–170. https://doi.org/10.1556/JBA.1.2012.006

Lozano, V., Soriano, M. F., Aznarte, J. I., Gómez-Ariza, C. J., & Bajo, M. T. (2016). Interference control commonalities in patients with schizophrenia, bipolar disorder, and borderline personality disorder. *Journal of Clinical and Experimental Neuropsychology*, *38*(2), 238–250. https://doi.org/10.1080/13803395.2015.1102870

Lutz, N. M., Chamberlain, S. R., Goodyer, I. M., Bhardwaj, A., Sahakian, B. J., Jones, P. B., & Wilkinson, P. O. (2022). Behavioral measures of impulsivity and compulsivity in adolescents with nonsuicidal self-injury. *CNS Spectrums*, *27*(5), 604–612. https://doi.org/10.1017/S1092852921000274

Machado, R. M., Kim, H. S., Oliveira de Andrade, V., Snaychuk, L. A., Moura, C. C., Belliero Martini, C., de Abreu, C. R. F. N., Hodgins, D. C., & Tavares, H. (2022). Gender differences in psychiatric comorbidity and personality characteristics among adults seeking treatment for problematic internet use. *Frontiers in Psychiatry*, *13*. https://doi.org/10.3389/fpsyt.2022.1022749

Martinelli, M., Chasson, G. S., Wetterneck, C. T., Hart, J. M., & Björgvinsson, T. (2014). Perfectionism dimensions as predictors of symptom dimensions of obsessive-compulsive disorder. *Bulletin of the Menninger Clinic*, *78*(2), 140–159. https://doi.org/10.1521/bumc.2014.78.2.140

Mazinan, R. G., Dudek, C., Warkentin, H., Finkenstaedt, M., Schröder, J., Musil, R., Kratzer, L., Fuss, J., & Biedermann, S. V. (2024). Borderline personality disorder and sexuality: causes and consequences of dissociative symptoms. *Borderline Personality Disorder and Emotion Dysregulation*, *11*(1), 8. https://doi.org/10.1186/s40479-024-00251-6

Mehrabian, A., & Russell, J. A. (1973). A Measure of Arousal Seeking Tendency. *Environment and Behavior*, *5*(3), 315–333. https://doi.org/10.1177/001391657300500303

Mellor, N. (2005). Attention seeking: The paradoxes of an under-researched concept. *Educational & Child Psychology*, *22*(4), 94–107.

Miller, C. E., Townsend, M. L., Day, N. J. S., & Grenyer, B. F. S. (2020). Measuring the shadows: A systematic review of chronic emptiness in borderline personality disorder. *PLoS ONE*, *15*(7 July). https://doi.org/10.1371/journal.pone.0233970

Mitchell, A. E., Dickens, G. L., & Picchioni, M. M. (2014). Facial Emotion Processing in Borderline Personality Disorder: A Systematic Review and Meta-Analysis. *Neuropsychology Review*, *24*(2), 166–184. https://doi.org/10.1007/s11065-014-9254-9

Moltu, C., Kverme, B., Veseth, M., & Natvik, E. (2023). How people diagnosed with borderline personality disorder experience relationships to oneself and to others. A qualitative in-depth study. *International Journal of Qualitative Studies on Health and Well-Being*, *18*(1). https://doi.org/10.1080/17482631.2022.2152220

Monk, J. K., Ogolsky, B. G., & Proulx, C. M. (2025). Clarifying relationship instability: Exploring the vital role of change in commitment. *Journal of Family Theory & Review*. https://doi.org/10.1111/jftr.12618

Moulding, R., Duong, A., Nedeljkovic, M., & Kyrios, M. (2017). Do You Think That Money Can Buy Happiness? A Review of the Role of Mood, Materialism, Self, and Cognitions in Compulsive Buying. *Current Addiction Reports*, *4*(3), 254–261. https://doi.org/10.1007/s40429-017-0154-y

Müller, A., Claes, L., Birlin, A., Georgiadou, E., Laskowski, N. M., Steins-Loeber, S., Brand, M., & De Zwaan, M. (2020). Associations of Buying-Shopping Disorder Symptoms with Identity Confusion, Materialism, and Socially Undesirable Personality Features in a Community Sample. *European Addiction Research*, *27*(2), 142–150. https://doi.org/10.1159/000511078

Müller, A., Claes, L., & Kyrios, M. (2021). Object attachment in buying-shopping disorder. *Current Opinion in Psychology*, *39*, 115–120. https://doi.org/10.1016/j.copsyc.2020.08.019

Müller, A., Georgiadou, E., Birlin, A., Laskowski, N. M., Jiménez-Murcia, S., Fernández-Aranda, F., Hillemacher, T., de Zwaan, M., Brand, M., & Steins-Loeber, S. (2022). The Relationship of Shopping-Related Decisions with Materialistic Values Endorsement, Compulsive Buying-Shopping Disorder Symptoms and Everyday Moral Decision Making. *International Journal of Environmental Research and Public Health*, *19*(7). https://doi.org/10.3390/ijerph19074376

Müller, A., Laskowski, N. M., Trotzke, P., Ali, K., Fassnacht, D. B., Zwaan, M. D. E., Brand, M., Hader, M., & Kyrios, M. (2021). Proposed diagnostic criteria for compulsive buying-shopping disorder: A Delphi expert consensus study. *Journal of Behavioral Addictions*, *10*(2), 208–222. https://doi.org/10.1556/2006.2021.00013

Müller, A., Müller, U., Albert, P., Mertens, C., Silbermann, A., Mitchell, J. E., & de Zwaan, M. (2007). Hoarding in a compulsive buying sample. *Behaviour Research and Therapy*, *45*(11), 2754–2763. https://doi.org/10.1016/j.brat.2007.07.012

Naguy, A. (2025). Borderline Personality Disorder and Self-Injurious Behaviours— Attention-Seeking or Attachment- Seeking? Psychopharmacological Considerations. *Psychopharmacology Bulletin*, *55*(2), 75–79. https://doi.org/10.64719/pb.4522

Ngo, T. T. A., Nguyen, H. L. T., Nguyen, H. P., Mai, H. T. A., Mai, T. H. T., & Hoang, P. L. (2024). A comprehensive study on factors influencing online impulse buying behavior: Evidence from Shopee video platform. *Heliyon*, *10*(15). https://doi.org/10.1016/j.heliyon.2024.e35743

Nica, E. I., & Links, P. S. (2009). Affective Instability in Borderline Personality Disorder: Experience Sampling Findings. *Current Psychiatry Reports*, *11*, 74–81. https://doi.org/10.1007/s11920-009-0012-2

Nicolai, J., & Moshagen, M. (2017). Dissociating pathological buying from obsessive-compulsive symptoms using delay discounting. *Zeitschrift Fur Psychologie / Journal of Psychology*, *225*(3), 244–251. https://doi.org/10.1027/2151-2604/a000308

Noguti, V., & Bokeyar, A. L. (2014). Who am I? The relationship between self-concept uncertainty and materialism. *International Journal of Psychology*, *49*(5), 323–333. https://doi.org/10.1002/ijop.12031

Norberg, M. M., Crone, C., Kwok, C., & Grisham, J. R. (2018). Anxious attachment and excessive acquisition: The mediating roles of anthropomorphism and distress intolerance. *Journal of Behavioral Addictions*, *7*(1), 171–180. https://doi.org/10.1556/2006.7.2018.08

Ociskova, M., Prasko, J., Hodny, F., Holubova, M., Vanek, J., Minarikova, K., Nesnidal, V., Sollar, T., Slepecky, M., & Kantor, K. (2023). Black & white relations: Intimate relationships of patients with borderline personality disorder. *Neuro Endocrinology Letters*, *44*(5), 321–331.

Paret, C., Jennen-Steinmetz, C., & Schmahl, C. (2017). Disadvantageous decision-making in borderline personality disorder: Partial support from a meta-analytic review. *Neuroscience and Biobehavioral Reviews*, *72*, 301–309. https://doi.org/10.1016/j.neubiorev.2016.11.019

Petersen, R., Brakoulias, V., & Langdon, R. (2016). An experimental investigation of mentalization ability in borderline personality disorder. *Comprehensive Psychiatry*, *64*, 12–21. https://doi.org/10.1016/j.comppsych.2015.10.004

Pfaltz, M. C., Passardi, S., Auschra, B., Fares-Otero, N. E., Schnyder, U., & Peyk, P. (2019). Are you angry at me? Negative interpretations of neutral facial expressions are linked to child maltreatment but not to posttraumatic stress disorder. *European Journal of Psychotraumatology*, *10*(1). https://doi.org/10.1080/20008198.2019.1682929

Price, A. L., Mahler, H. I. M., & Hopwood, C. J. (2022). Construction and Validation of a Self-report Subjective Emptiness Scale. *Assessment*, *29*(3), 397–409. https://doi.org/10.1177/1073191120968275

Raemen, L., Luyckx, K., Müller, A., Buelens, T., Verschueren, M., & Claes, L. (2020). Non-Suicidal Self-Injury and Pathological Buying in Community Adults and Patients with Eating Disorders: Associations with Reactive and Regulative Temperament. *Psychologica Belgica*, *60*(1), 396–410. https://doi.org/10.5334/pb.1027

Reeves, R. A., Baker, G. A., & Truluck, C. S. (2012). Celebrity Worship, Materialism, Compulsive Buying, and the Empty Self. *Psychology & Marketing*, *29*(9), 674–679. https://doi.org/10.1002/mar.20553

Ross, S. R., Keiser, H. N., Strong, J. V., & Webb, C. M. (2013). Reinforcement sensitivity theory and symptoms of personality disorder: Specificity of the BIS in Cluster C and BAS in Cluster B. *Personality and Individual Differences*, *54*(2), 289–293. https://doi.org/10.1016/j.paid.2012.09.020

Scherhorn, G., Reisch, L. A., & Raab, G. (1990). Addictive Buying in West Germany: An Empirical Study. *Journal of Consumer Policy*, *14*, 355–387.

Sebastian, A., Jacob, G., Lieb, K., & Tüscher, O. (2013). Impulsivity in borderline personality disorder: A matter of disturbed impulse control or a facet of emotional dysregulation? *Current Psychiatry Reports*, *15*(2). https://doi.org/10.1007/s11920-012-0339-y

Selby, E. A., Anestis, M. D., Bender, T. W., & Joiner, T. E. (2009). An exploration of the emotional cascade model in borderline personality disorder. *Journal of Abnormal Psychology*, *118*(2), 375–387. https://doi.org/10.1037/a0015711

Serfas, B. G., Büttner, O. B., & Florack, A. (2014). Eyes Wide Shopped: Shopping Situations Trigger Arousal in Impulsive Buyers. *PLoS ONE*, *9*(12), e114593. https://doi.org/10.1371/journal.pone.0114593

Sharif, S. P., & Khanekharab, J. (2017). Identity Confusion and Materialism Mediate the Relationship between Excessive Social Network Site Usage and Online Compulsive Buying Journal: Cyberpsychology, Behavior, and Social Networking. *Cyberpsychology, Behavior, and Social Networking*, *20*(8), 494–500. https://doi.org/10.1089/cyber.2017.0162

Sherman, J. A., & Ehrenreich-May, J. (2017). Ethnicity’s Role in the Relationship Between Anxiety and Negative Interpretation Bias Among Clinically Anxious Youth: A Pilot Study. *Child Psychiatry & Human Development*. https://doi.org/10.1007/s10578-017-0760-x

Smith, M., & South, S. (2020). Romantic attachment style and borderline personality pathology: A meta-analysis. *Clinical Psychology Review*, *75*, 101781. https://doi.org/10.1016/j.cpr.2019.101781

Soloff, P. H., Abraham, K., Ramaseshan, K., Burgess, A., & Diwadkar, V. A. (2017). Hyper-modulation of brain networks by the amygdala among women with Borderline Personality Disorder: Network signatures of affective interference during cognitive processing. *Journal of Psychiatric Research*, *88*, 56–63. https://doi.org/10.1016/j.jpsychires.2016.12.016

Stead, V. E., Boylan, K., & Schmidt, L. A. (2019). Longitudinal associations between non-suicidal self-injury and borderline personality disorder in adolescents: a literature review. *Borderline Personality Disorder and Emotion Dysregulation*, *6*(1), 3. https://doi.org/10.1186/s40479-019-0100-9

Stoeber, J. (2014). Multidimensional perfectionism and the DSM-5 personality traits. *Personality and Individual Differences*, *64*, 115–120. https://doi.org/10.1016/j.paid.2014.02.031

Theisejans, J., Thomas, T. A., Oueslati, F. B. V., Kessling, A., Wegmann, E., Müller, A., & Brand, M. (2025). Affective and Cognitive Functions, Impulsivity and Compulsivity in Problematic Social Network Use and in Compulsive Buying-Shopping Disorder: A Systematic Review. *Current Addiction Reports*, *12*(1). https://doi.org/10.1007/s40429-025-00688-x

Tiffany, S. T., & Wray, J. M. (2012). The clinical significance of drug craving. *Annals of the New York Academy of Sciences*, *1248*(1), 1–17. https://doi.org/10.1111/j.1749-6632.2011.06298.x

Timpano, K. R., & Port, J. H. (2021). Object attachment and emotion (Dys)regulation across development and clinical populations. *Current Opinion in Psychology*, *39*, 109–114. https://doi.org/10.1016/j.copsyc.2020.08.013

Topino, E., Cacioppo, M., & Gori, A. (2022). The Relationship between Attachment Styles and Compulsive Online Shopping: The Mediating Roles of Family Functioning Patterns. *International Journal of Environmental Research and Public Health*, *19*(13), 8162. https://doi.org/10.3390/ijerph19138162

Trotzke, P., Starcke, K., Müller, A., & Brand, M. (2019). Cue-induced craving and symptoms of online-buying-shopping disorder interfere with performance on the Iowa Gambling Task modified with online-shopping cues. *Addictive Behaviors*, *96*, 82–88. https://doi.org/10.1016/j.addbeh.2019.04.008

Trotzke, P., Starcke, K., Pedersen, A., & Brand, M. (2014). Cue-induced craving in pathological buying: Empirical evidence and clinical implications. *Psychosomatic Medicine*, *76*(9), 694–700. https://doi.org/10.1097/PSY.0000000000000126

Trotzke, P., Starcke, K., Pedersen, A., Müller, A., & Brand, M. (2015). Impaired decision making under ambiguity but not under risk in individuals with pathological buying-behavioral and psychophysiological evidence. *Psychiatry Research*, *229*(1–2), 551–558. https://doi.org/10.1016/j.psychres.2015.05.043

Van Malderen, E., Goossens, L., Claes, L., Wilderjans, T. F., Kemps, E., & Verbeken, S. (2024). Self-regulation profiles in addictive behaviors among adolescents: A transdiagnostic approach. *Appetite*, *192*. https://doi.org/10.1016/j.appet.2023.107128

Varvaras, J., Schaar, P., Laskowski, N. M., Georgiadou, E., Norberg, M., & Müller, A. (2025). To hoard or not to hoard purchased items - does it matter? A clinical cross-sectional study on compulsive buying-shopping disorder. *Comprehensive Psychiatry*, 152588. https://doi.org/https://doi.org/10.1016/j.comppsych.2025.152588

Verplanken, B., & Sato, A. (2011). The Psychology of Impulse Buying: An Integrative Self-Regulation Approach. *Journal of Consumer Policy*, *34*(2), 197–210. https://doi.org/10.1007/s10603-011-9158-5

Victor, S. E., Glenn, C. R., & Klonsky, E. D. (2012). Is non-suicidal self-injury an “addiction”? A comparison of craving in substance use and non-suicidal self-injury. *Psychiatry Research*, *197*(1–2), 73–77. https://doi.org/10.1016/j.psychres.2011.12.011

Villardefrancos, E., & Otero-López, J. M. (2016). Compulsive buying in university students: Its prevalence and relationships with materialism, psychological distress symptoms, and subjective well-being. *Comprehensive Psychiatry*, *65*, 128–135. https://doi.org/10.1016/j.comppsych.2015.11.007

Vogt, S., Hunger, A., Pietrowsky, R., & Gerlach, A. L. (2015). Impulsivity in consumers with high compulsive buying propensity. *Journal of Obsessive-Compulsive and Related Disorders*, *7*, 54–64. https://doi.org/10.1016/j.jocrd.2015.10.002

Voth, E. M., Claes, L., Georgiadou, E., Selle, J., Trotzke, P., Brand, M., De Zwaan, M., & Müller, A. (2014). Reactive and regulative temperament in patients with compulsive buying and non-clinical controls measured by self-report and performance-based tasks. *Comprehensive Psychiatry*, *55*(7), 1505–1512. https://doi.org/10.1016/j.comppsych.2014.05.011

Wegmann, E., Müller, S. M., Kessling, A., Joshi, M., Ihle, E., Wolf, O. T., & Müller, A. (2023). Online compulsive buying-shopping disorder and social networks-use disorder: More similarities than differences? *Comprehensive Psychiatry*, *124*. https://doi.org/10.1016/j.comppsych.2023.152392

Wilkinson-Ryan, T., & Westen, D. (2000). Identity Disturbance in Borderline Personality Disorder: An Empirical Investigation. *American Journal of Psychiatry*, *157*(4), 528–541. https://doi.org/10.1176/appi.ajp.157.4.528

Winter, D., Herbert, C., Koplin, K., Schmahl, C., Bohus, M., & Lis, S. (2015). Negative evaluation bias for positive self-referential information in borderline personality disorder. *PLoS ONE*, *10*(1). https://doi.org/10.1371/journal.pone.0117083

Winter, D., Koplin, K., Schmahl, C., Bohus, M., & Lis, S. (2016). Evaluation and memory of social events in borderline personality disorder: Effects of valence and self-referential context. *Psychiatry Research*, *240*, 19–25. https://doi.org/10.1016/j.psychres.2016.03.042

Workman, L., & Paper, D. (2010). Compulsive Buying: A Theoretical Framework. *The Journal of Business Inquiry*, *9*, 1. http:www.uvu.edu/woodbury/jbi/volume9

World Health Organization. (2022). *International Classification of Diseases 11th Revision*. https://icd.who.int/en

Zanarini, M. C., Reichman, C. A., Frankenburg, F. R., Reich, D. B., & Fitzmaurice, G. (2010). The course of eating disorders in patients with borderline personality disorder: A 10‐year follow‐up study. *International Journal of Eating Disorders*, *43*(3), 226–232. https://doi.org/10.1002/eat.20689

Zerach, G. (2016). The Mediating Role of Emptiness and Materialism in the Association Between Pathological Narcissism and Compulsive Buying. *International Journal of Mental Health and Addiction*, *14*(4), 424–437. https://doi.org/10.1007/s11469-015-9591-9

Zuckerman, M. (1994). *Behavioral expressions and biosocial bases of sensation seeking*. Cambridge University Press.
